# Assessing the Effect of Specimen Preparation Methods on DSR Test Results of Bitumen Using Factorial Design Analysis

**DOI:** 10.3390/ma17205117

**Published:** 2024-10-19

**Authors:** Maya Sheidaei, Jiqing Zhu, Sven Agardh

**Affiliations:** 1 Department of Technology and Society, Lund University, P.O. Box 118, 22100 Lund, Sweden; sven.agardh@tft.lth.se; 2 Swedish National Road and Transport Research Institute (VTI), 58330 Linköping, Sweden; jiqing.zhu@vti.se

**Keywords:** bitumen, dynamic shear rheometer (DSR), shear modulus, rheology, specimen preparation, phase angle

## Abstract

A two-level three-factor factorial design experiment was conducted to study the influences of three critical specimen preparation parameters on the measurement results of bitumen by a dynamic shear rheometer (DSR). The investigated factors were (1) the pre-heating temperature (HT) for manufacturing the specimen, (2) the bonding temperature (BT) onto the rheometer, and (3) the trimming (Trim) operation for preparing the specimen after bonding. The analysed data were the measured shear modulus |G*|, phase angle *δ*, and the characteristic temperatures of bitumen’s specific stiffness *TX* with corresponding phase angle *δ_TX_* according to the European standard EN 14770:2023. Five types of bitumen were tested, including three penetration grades and two modified bitumen specimens (with polymer and wax additives). In addition, a repeatability evaluation of the test results was conducted. We found that the trimming operation for preparing the specimen has a noticeable impact when using smaller plates (PP08) for the DSR measurement. At higher test temperatures when using larger plates (PP25), the trimming operation does not significantly impact the measured parameters, in contrast to the HT and BT. Except for bitumen type 70/100, modified binders are more susceptible to variation in the analysed parameters than unmodified ones. The three-way interaction Trim:BT:HT tends to cause relatively little variation in measured data. Interactions between two factors Trim:BT, Trim:HT, and BT:HT contribute more to the fluctuation in *δ* value than in *TX* and |G*|. The variation employed in this study affects the test repeatability of wax-modified bitumen significantly; however, for unmodified binders the repeatability of *TX* and *δ_TX_* are within 0.4–2.1 °C and 0.3–3.1°, respectively.

## 1. Introduction

The dynamic shear rheometer (DSR) measures the rheological properties of bituminous binders as a function of loading time and temperature rather than empirical parameters. The complex shear modulus is obtained from DSR testing, defined as G* = |G*| e*^i^^δ^*, where |G*| is the dynamic shear modulus, *δ* is the phase angle, and *i* is the imaginary unit (*i*^2^ = −1). The G* represents the specimen’s total resistance to deformation when sheared repeatedly, and the phase angle (*δ*) represents the lag between the applied shear stress (*τ*) and the generated shear strain (*γ*). The results also can be expressed at temperatures *TX* at which |G*| has a specific value and corresponding phase angles (*δ_TX_*). The characteristic value *T*(|G*| = 5 MPa) represents the intermediate service temperature, *T*(|G*| = 15 kPa) represents the high service temperature, and *T*(|G*| = 1 kPa) represents the upper limit temperature suitable for testing with the DSR 25 mm plates (PP25) according to EN 14770:2023 [1]. However, modern rheometers can achieve an acceptable measuring result at very low torque in the case of material with low viscosity at higher temperatures. The 4 mm parallel plate and cone-and-plate methods can also be used in DSR testing to explain the relaxation properties of bituminous binders [2,3], which are not covered in this study.

In Europe, penetration-based bitumen specification is more common than the performance-related specification based on DSR test results for the asphalt industry in practice, despite the research interest in DSR testing to ease the transition [4]. The standardised procedure of preparing the test sample and specimens for DSR testing is mainly described in the methods EN 12594:2014 [5] and EN 14770:2023 [1]. However, various studies have shown that factors, such as plate diameter and/or gap size variation [6,7,8,9,10,11], the specimen manufacturing method [12], overfilling or underfilling of the space between plates [13,14], and the limit of linear viscoelasticity [15], affect the consistency of the test results. Additionally, the effects of the equipment’s sensitivity in measuring torque [16], the pre-heating temperature for manufacturing the specimen [17], and the storage duration of the manufactured specimen before the test start [18] have also been investigated. Furthermore, other research investigations have pointed out the importance of having the same thermal treatment to achieve acceptable precision and comparable results during the specimen preparation and testing phase, once the specimen is mounted on the rheometer [19,20].

A variety of factors, according to the previous research, can influence the measured rheological properties of bitumen using a DSR. However, most of the research has focused on only one variable while holding all other variables constant, making it difficult to compare results and comprehend the interplay between factors. This research aims to assess if (1) the pre-heating temperature (HT) for manufacturing specimens in a mould, (2) the bonding temperature (BT) at which the specimen bonds onto the DSR, and (3) the radial trimming (Trim) operation of the specimen have any significant impact on the outcome and under what conditions they may affect. The factors were selected based on a prior screening of data from a set of interlaboratory tests among European laboratories [11]. This screening study investigated the equipment brand, specimen manufacturing technique, storage duration of specimens, pre-heating temperature and duration for manufacturing specimens, the temperature at which the specimen bonds to the DSR, equilibrium duration, and linear viscoelastic range. Our findings demonstrated that, compared to other steps, the bonding temperature (BT) had a small but significant impact on more tested conditions. Additionally, the pre-heating temperature (HT) of bitumen used to manufacture the specimen must be carefully selected. Furthermore, the radial trimming (Trim) of excess material, which represents the largest intervention to the specimen, was of interest. In addition, this paper also analysed the repeatability and the interaction effects between the three studied factors. 

## 2. Materials and Methods

Table 1 summarises the basic features of the five different binders tested in this study. The results of the needle penetration test (PEN) per EN 1426:2015 [21], the softening point (SP) test per EN 1427:2015 [22], and the bitumen density at 25 °C per ASTM D70:2008 [23], which is used to calculate the amount of bitumen required for specimen preparation, are provided by the material supplier, Peab Asfalt in Gothenburg, Sweden. Kraton D1192, a linear styrene-butadiene-styrene (SBS) block copolymer with bound styrene of 30% by mass and a molecular weight of 138–162 kg/mol, and Sasobit, a long-chain aliphatic hydrocarbon synthetic prill form with a 5 mm diameter which is sulfur-free with a congealing point of 100–110 °C and produced by Sasol Wax in South Africa are the two additives that are used to modify bitumen. These additives are used worldwide to improve the quality and durability of asphalt roads. SBS-modified bitumen increases its resistance to fatigue and permanent deformation while also improving its elastic recovery, in addition to increasing its softening point and decreasing its penetration [24]. Sasobit wax lowers the blending temperatures of asphalt mix manufacturing and enhances rutting resistance by stiffening the bitumen [25]. The bitumen 70/100 samples were mixed by weight of 23% of the 160/220 and 77% of the 50/70 for each set of 8 combinations separately, which appeared to have influenced the contribution of effects on outcomes differently from other tested materials. The same bitumen 70/100 was used to blend with SBS and wax (same batch for all combinations), and the additive content was 4% for both types.

### 2.1. Design of Experiments

This experiment is designed to identify the effect of each of the three factors, as well as the effect of interactions between factors on the DSR testing outcomes (|G*|, *δ*, *TX*, and *δ_TX_*) rather than creating a statistical model to predict the outcomes. All 8 different combinations of the 2^3^ factorial design used in this study are shown in Table 2, where the three following factors are each assigned two levels: the specimen’s radial trimming operation (Trim), the DSR plates’ temperature at which specimen was bonded (BT), and the temperature at which bitumen was pre-heated to purify into a mould to manufacture the specimen (HT). The high (+1) and low (−1) levels for trimming were set as not trimmed (No) and trimmed (Yes); the BT levels were SP (−1) of the tested materials and SP + 25 °C (+1), and the HT levels were SP + 80 °C (−1) and SP + 100 °C (+1), respectively. The existing standard method EN 14770:2023 was taken into consideration for the allocation of high and low levels for each of these factors. The expected SP of bitumen plus 5 °C to 20 °C is recommended as the bonding temperature, which was expanded by 5 °C in this study to make the potential effect more evident. The upper limits of 100 °C above the expected SP for unmodified bitumen and 180 °C to 200 °C for modified bitumen are recommended by the standard as the heating temperatures; however, a lower temperature was chosen to investigate its sensitivity. Two replicates were carried out for each of the 8 combinations. To ensure that variation testing was carried out under the same experimental conditions, a random testing order was assigned for each of the 16 tests (Table 2).

DSR rheological measurements yield |G*| and *δ*. With the measurement results, certain calculations on the |G*| and *δ* were conducted to obtain values of temperature *TX* and the related phase angle *δ_TX_* for particular levels of the dynamic shear modulus |G*| at 5 MPa, 15 kPa, and 1 kPa with an appropriate parallel plate geometry (PP08 or PP25) at a frequency of 10 rad/s (1.59 Hz). The temperatures *TX* were calculated using logarithmic interpolation from two distinct data points that are no more than 10 °C apart (Table 3). The equivalent phase angle *δ_TX_* was calculated using linear interpolation from the same data points. Temperatures at which specimens were trimmed, if listed, are shown in bold in Table 3. Specimens without bold format indicate that trimming temperatures differ by 10 °C from the tested temperatures.

### 2.2. Preparation of Specimens and DSR Test Setup

An Anton Paar MCR302 dynamic shear rheometer with RheoCompass software was used for measuring the shear modulus and phase angle of bitumen in an oscillatory-type testing mode. To ensure conditions remained in the linear viscoelastic (LVE) range, amplitude sweep (A-sweep) tests were performed at each test temperature according to the current standard (EN 14770:2023). Strain amplitude limits of 0.5% (0.005 mm/mm) for 70/100, 0.1% for 70/100 + 4% SBS, and 0.05% for 70/100 + 4% Wax were suitable for the tested temperatures. For bitumen 50/70 and 160/220, the strain amplitude limits of 0.5% and 0.1% were used with PP25 and PP08, respectively. The 2^3^ (2-level 3-factor) factorial design with two replicates leads to 16 tests in total. Three specimens were used for each of the tests. One specimen was tested (in descending order of temperature) using PP08, and the other two specimens were tested using PP25 (in ascending order of temperature). The same operator performed all 48 temperature sweeps (T-sweep) per material type in total, with two repeats over the 8 combinations that involved three specimens each. A 15-min thermal equilibration time was used to ensure that the temperature in the test specimen did not differ from the temperature recorded by the DSR. Consequently, the gap-setting, loading the DSR with bitumen, A-sweep, and T-sweep testing took over 1 h per specimen. To eliminate the potential impact of static hardening, the manufactured specimens were covered with an opaque lid and held at room temperature for 2 to 20 h before the test procedure began. For all materials and tests, Specimen 1 (PP08) was stored for 2 h, Specimen 2 (PP25) for no longer than 10 h, and Specimen 3 (PP25) for no more than 20 h. Using the same relative storage time, the possible hardening effect was removed from the analysis.

The specimens were manufactured in silicone moulds after pre-heating the material at the planned temperatures (either SP + 80 °C or SP + 100 °C). In the cases without trimming, the exact amount of bitumen material was calculated based on the volume of the specimen and the density of the binder, resulting in a bulge around the periphery while testing. Meanwhile, the amount of bitumen placed on the DSR for preparing the specimen with trimming was somewhat higher than without trimming. The zero-gap setting before loading the DSR with bitumen was carried out immediately after the first planned test temperature was applied and then again 10 min after the plates reached the applied temperature. After that, the planned bonding temperatures (either at SP + 0 °C or SP + 25 °C) were applied, and the temperature was held for 10 min once the bonding temperature was reached. In the case of trimming, the manufactured specimen was placed on the DSR’s lower plate, and the gap was reduced to either 2 + 0.10 mm (PP08) or 1 + 0.05 mm (PP25) for trimming. A spatula was pre-heated and used to trim the specimen. Then, the gap was finalised at 2 mm or 1 mm depending on the plate size and the testing was started. To prevent variations in measured |G*| and *δ* due to varying amounts of surplus bitumen across different tests, the same amount of bitumen was used throughout the experiment. Also, scorching the spatula was avoided, which could cause smoking during trimming. The trimming procedure was carried out in a few stages, and the excess bitumen was wiped away from the spatula after each stage.

## 3. Statistical Analysis

The factorial design described above was used to generate coefficients for a linear model that is a function of various components and predicts the actual system outcome (Equation (1)). The model contains all interaction terms regardless of their significance. However, rather than developing a mathematical model for the system, this study is mainly concerned with discovering general trends and the influence of various components on system results.

(1) $${\hat{Y}}_{i\left(\dot{i}:1-8\right)}=a_{0}+{(a}_{1})\cdot Trim+{(a}_{2})\cdot BT+{(a}_{3})\cdot HT+(a_{4})\cdot Trim\cdot BT+(a_{5})\cdot Trim\cdot HT+{(a}_{6})\cdot BT\cdot HT+\;{(a}_{7})\cdot Trim\cdot BT\cdot HT$$

Here, ${\hat{Y}}_{i}$ is a modelled outcome of the *i:th* combination, the intercept $a_{0}$ is the mean of all combinations, and the $a_{i}$ is the effect coefficient that is estimated using the least square method in the R software package [26], which can also be calculated from the contrasts between combinations in the factorial design [27]. The coefficients obtained using the contrast between combinations are twice as large as those calculated using the least squares approach in R. This is due to the calculated effects yielding changes of two units along each axis from (−1) to (+1). Three main effects ($\frac{N!}{1!\left(N-1\right)!}$), three two-way interactions ($\frac{N!}{2!\left(N-2\right)!}$), and one three-way interaction ($\frac{N!}{3!\left(N-3\right)!}$) were estimated using a 2^N=3^ full factorial design (2-level 3-factor) with two replicates (Equation (1)). Three-way interactions are not simple to conceptualise. The basic interaction between any two given factors differs depending on the degree of the third factor. Since we know that all three factors influence the results, the three-factor interaction is not of interest in this experiment. However, since there is only one three-way interaction, it was not particularly sensible to explore lower-order interactions using a fractional factorial design rather than a full factorial design. 

The statistical analysis was performed using the R software package [26] to calculate the error variance and standard errors of the effects from replicated tests on measured |G*|, *δ*, *TX*, and *δ_TX_*, as shown in the Results section. The analysis of variance (ANOVA) method is used to determine whether there are statistically significant differences between the means of the combinations. An effect (main or interaction) is considered as statistically significant if the probability level that the F-statistic calculated from the data is less than 5%, at which point the null hypothesis could be rejected. In this study, the hypothesis is that the mean yield when the factors are set at the high level (+1) varies from when the factors are set at the low level (−1). As a result, the null (*H*_0_) and alternative (*H*_1_) hypotheses can be expressed as shown in Equation (2).

(2) $$H_{0}:a_{i}=O\left(\mu_{+1}=\mu_{-1}\right)\;H_{1}:a_{i}\neq O\left(\mu_{+1}\neq \mu_{-1}\right)$$

As an example (Equation (3)), the following reckoning is a model for *T*(|G*| = 5 MPa) per Equation (1). It represents the outcome of the experimental design and the eight combinations that were conducted for bitumen 70/100 at 10 rad/s. Table 4 provides the analysis of variance of computed data using the R software. The asterisk indicates statistical significance at a level of 5%.

(3) $${\hat{T}}_{i\left(\dot{i}:1-8\right)}\left(\left|G^{*}\right|=5\;MPa\right)=12.99+{\left(0.245\right).Trim}_{i}+\left(-0.014\right){.BT}_{i}+\left(0.136\right){.HT}_{i}+\left(-0.062\right).{Trim}_{i}\cdot {BT}_{i}+\;\left(-0.028\right).{Trim}_{i}\cdot {HT}_{i}+\left(-0.108\right).{BT}_{i}\cdot {HT}_{i}+\left(-0.446\right).{{Trim}_{i}\cdot BT}_{i}\cdot {HT}_{i}$$

**Figure 1:**
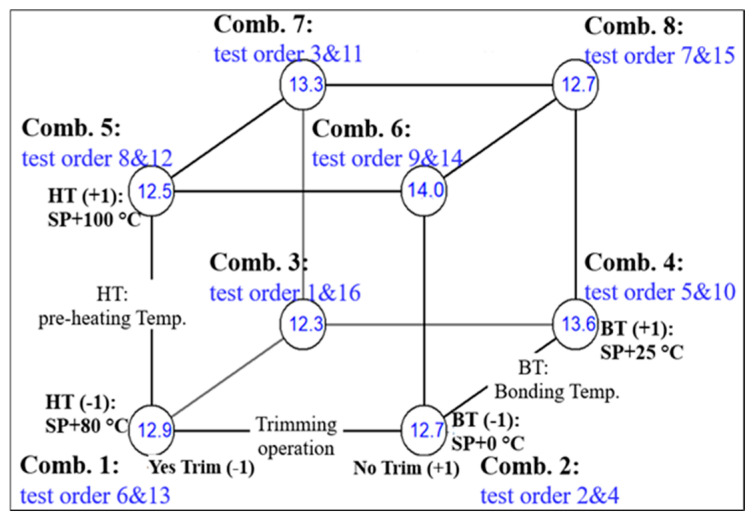
Cube plot for the outcome *T*(|G*| = 5 MPa) for bitumen 70/100. The averages of replicated test results are shown inside the circles.

For convenience, the factorial design analysis of effects from the contrasts between combinations is illustrated just for the outcome *T*(|G*| = 5 MPa) of bitumen 70/100 in Figure 1; however, the approach for other outcomes and binders is similar. Figure 1 displays the average test results for each combination, as well as the testing orders. 

The data values obtained for |G*|, *δ*, *TX*, and *δ_TX_* are averaged over two replicates. The main effect is the difference between averages of four outcomes (Ȳ) for which the effect under study is at its high and low levels, for instance, Trim(*Y*) = (Ȳ_Trim+1_ − Ȳ_Trim−1_). As for the measure of a two-way interaction, for instance, Trim.HT is the difference between the average trimming effects (Trim) at the high and low levels of HT. The three-way interaction Trim.BT.HT is defined as the average difference between the Trim.BT interaction at the two different levels of HT. Table 5 shows the results of applying the described approach to conduct a factorial design analysis of effects on *T*(|G*| = 5 MPa) of bitumen 70/100. The asterisk indicates a statistically significant effect (Trim.BT.HT) at a level of 5%.

The standard error (SE) of an effect is the square root of the average of the estimated variance across the 8 combinations for an effect, which itself is the difference between the average of the high and low levels (Table 5).

## 4. Results and Discussion

### 4.1. Repeatability Evaluation of Test Results

The mean values were calculated from the eight different combinations for each material (Table 6 and Table 7).

The experimental results covering the eight different test combinations were used to estimate the repeatability with 95% confidence r (=2.77*s_r_), where s_r_ is the repeatability standard deviation defined as Equation (4), for *TX* and *δ_TX_* and |G*| and *δ* measured with PP08 and PP25 plates. In Equation (4), the *Y*1*_i_* and *Y*2*_i_* are two replicates of the i:th combination of the measured outcomes.

(4) $$s_{r}\;(Y)=\sqrt{\sum_{i=1}^{i=8}{Var(Y1}_{i}\&{Y2}_{i})∕8}$$

**Figure 2:**
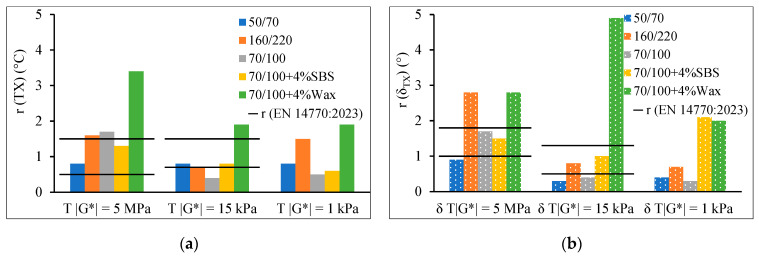
Estimated r (2.77*s_r_): (**a**) *TX* (°C), (**b**) *δ_TX_* (°).

**Figure 3:**
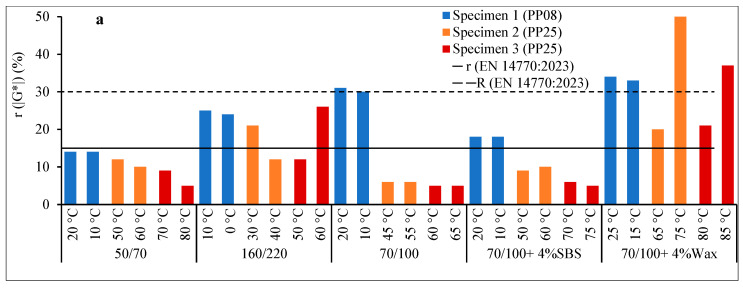

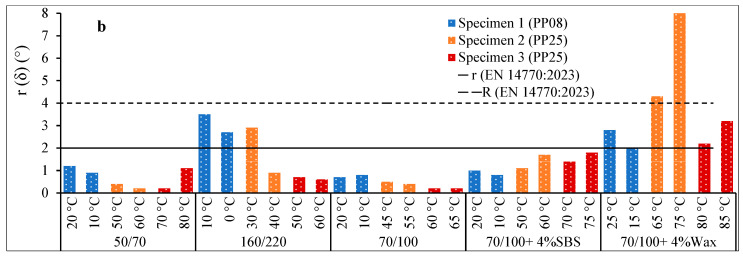
Estimated r (2.77*s_r_) at tested pair temperatures according to the testing plan: (**a**) |G*| (%), (**b**) *δ* (°).

The estimates of the repeatability r for the |G*| and *δ* listed in EN 14770:2023 are 15% and 2°, respectively. The values given are independent of the type of bitumen, state of bitumen, specimen geometry, and test temperatures and frequencies. The repeatability results, r, in the standard for *T*(|G*| = 5 MPa) and *δT*(|G*| = 5 MPa) measured with PP08 are within 0.5–1.5 °C and 1.0–1.8°, respectively, while for *T*(|G*| = 15 MPa) and *δT*(|G*| = 15 MPa) measured with PP25 are within 0.7–1.5°C and 0.5–1.3°, respectively. Figure 2 and Figure 3 show how significantly the variations employed in specimen preparation, when testing with the DSR, affected the repeatability of wax-modified bitumen. The variations in examined factors influenced the repeatability of modified bitumen as follows: 0.6–3.4 °C for r(*TX*), 1.0–4.9° for r(*δ_TX_*), 6–34% for r(|G*|), and 1.0–4.3° for r(δ). Meanwhile, for unmodified bitumen, the repeatability values are within 0.4–2.1 °C for r(*TX*), 0.3–3.1° for r(*δ_TX_*), 5–31% for r(|G*|), and 0.2–3.5° for r(*δ*) (Figure 2). The estimated repeatability r for dynamic shear modulus |G*| and phase angle *δ* is higher than the given values in EN 14770:2023. However, considering the different testing conditions for each combination in this research, it is below and/or within the given repeatability (r) and reproducibility (R) boundaries for most of the tested temperatures (Figure 3). Note that the R limit is according to the standard. No information is given for the reproducibility of the current single laboratory study, as reproducibility describes the precision between laboratories.

### 4.2. Affecting Factors

According to the analysis of variance, factors with larger sums of squares values have greater effects on the outcomes than other factors that are more consistent with results obtained via contrasts between combinations. Due to space limitations, only the estimated effects and their SE values for the outcomes (|G*|, *δ*, *TX*, and *δ_TX_*) are shown in Table 8 and Table 9. This involves comparing the data that were generated for the characteristic values *TX* and corresponding *δ_TX_*. Additionally, the estimated effects and their SEs on the measured |G*| and *δ* are provided during testing at temperatures below and around the binders’ softening points. The factors that have a statistically significant impact on the outcomes of the tested materials were identified, where the *p*-values from the ANOVA test were less than the confidence limit (α = 0.05).

Table 8 and Table 9 reveal that the *TX* has more effects that are statistically significant than the *δ_TX_* in terms of the total numbers of significant effects detected (42 and 26, respectively). However, comparing the SE reveals that the outcome phase angles (*δ_TX_* and *δ*) for modified bitumen vary more than they do for unmodified ones, with ranges of 0.18–0.88 for modified bitumen and 0.03–0.63 for unmodified bitumen. This could be due to the modified bitumen being more sensitive to variations in testing conditions than the unmodified bitumen. For instance, compared to unmodified bitumen, the homogeneity of the manufactured samples may have differed significantly depending on the heating temperature of SP + 80 °C or SP + 100 °C [28]. The chosen high and low levels conditions of the studied factors are relatively close to each other; thus, very few significant effects were expected. Nevertheless, *TX* may be a better option for result expression than |G*| because it is determined from two separate data points, which could account for the increased consistency across tested materials. 

The results show that when PP08 was used, the *T*(|G*| = 5 MPa) values increased significantly with the technique of preparing specimens without trimming as compared to trimming the specimens (Table 8). Specimens that are not trimmed also have higher measured |G*| values (Table 9). This significant effect of the trimming operation at low temperatures may be attributed to the fact that PP08 is harder to trim than PP25 because of its smaller plate diameter and that the bitumen thickness in PP08 is twice as thick (2 vs. 1 mm), which can cause the bitumen sample to shrink twice as much when it cools. However, the short temperature range tested per specimen lowers the possibility of volume change in the specimen as temperature changes. Except for bitumen 70/100 and 70/100 + 4% SBS, there was no significant difference in the *T*(|G*| = 15 kPa) and *T*(|G*| = 1 kPa) values between the trimming and non-trimming techniques when PP25 was used for the testing. Trimming the specimen in the case of SBS-modified bitumen was difficult, even in the case of PP25, which is easier than PP08 due to its specimen shape. The bitumen was rubberised, and a small amount of bitumen was pulled out or an excess amount of barely noticeable bitumen became stuck around the outside perimeter of the DSR plates during trimming, while we attempted to avoid overheating the spatula for trimming. According to a study [29], repeatability can be enhanced by methodically controlling the temperature of the trimming tool and using a single operator; as a result, variance between the trimmed samples is minimal in this study.

Interestingly, the bonding temperatures alone (either low or high) did not influence *TX* values, but combined with trimming, the effect became statistically significant—higher *TX* values were recorded when the specimen was bonded in SP + 25 °C and not trimmed. The HT and BT appeared to have greater effects than the trimming operation on measured outcomes when tested at intermediate and higher temperatures with PP25. According to the coefficient of main effect BT, increasing the bonding temperature from SP (low level) to SP + 25 °C (high level) decreased the measured values of *δ_T_*_(|G*|=5MPa)_ and *δ_T_*_(|G*|=15kPa)_ for almost all the tested materials. This means higher elastic behaviour of the bitumen could be concluded when bonded at a higher temperature than at a lower temperature. For all tested materials, the effect of HT appeared to be minimal on *δ_T_*_(|G*|=5MPa)_. As for the wax-modified bitumen, the HT had a substantial effect on the measured *δ* at higher test temperatures, resulting in a lower *δ_T_*_(|G*|=1kPa)_ value with a higher heating temperature SP + 100 °C compared to the lower heating temperature SP + 80 °C. However, because the material was not subjected to extremely high temperatures or an extended duration of heating, the increased viscous behaviour at higher test temperatures cannot be attributed to the aging of bitumen. The findings for |G*| and *δ* are consistent with previous study [30]. The greater effect of the HT (SP + 80 °C or SP + 100 °C) on measured outcomes when tested at intermediate and higher temperatures with PP25 may be related to bitumen morphology, which differs greatly from tests performed at lower temperatures. When Xie et al. [31] conducted tests at temperatures below and around the SP of the studied materials (25 °C to 65 °C), they observed a strong correlation between the bitumen morphology and mechanical property (i.e., modulus). They found that bitumen’s surface micromorphology, which varies depending on the type and composition of bitumen (e.g., paraffin wax), was obviously influenced by temperature.

**Figure 4:**
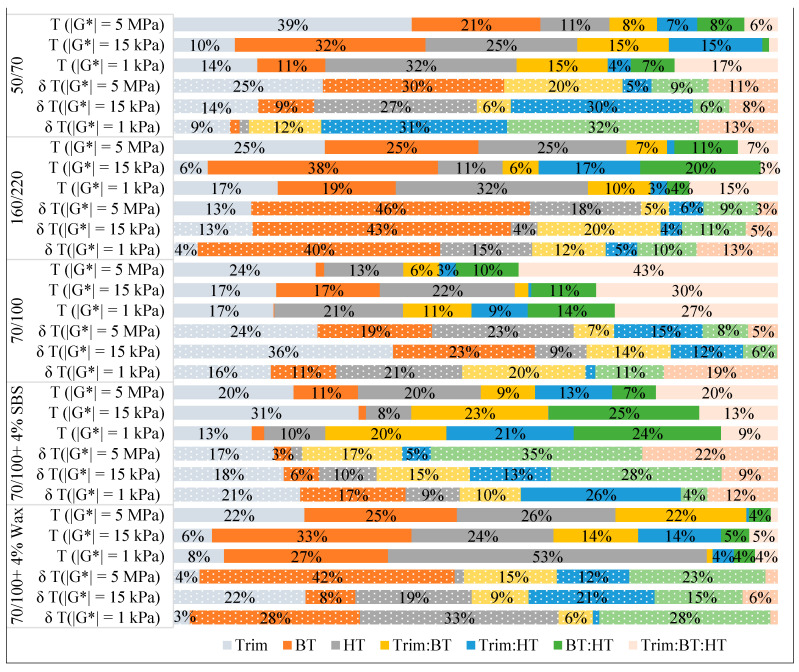
The stacked bar chart shows the effect contributions in % to the variations in *TX* (solid-filled) and corresponding *δ_TX_* (pattern-filled).

**Figure 5:**
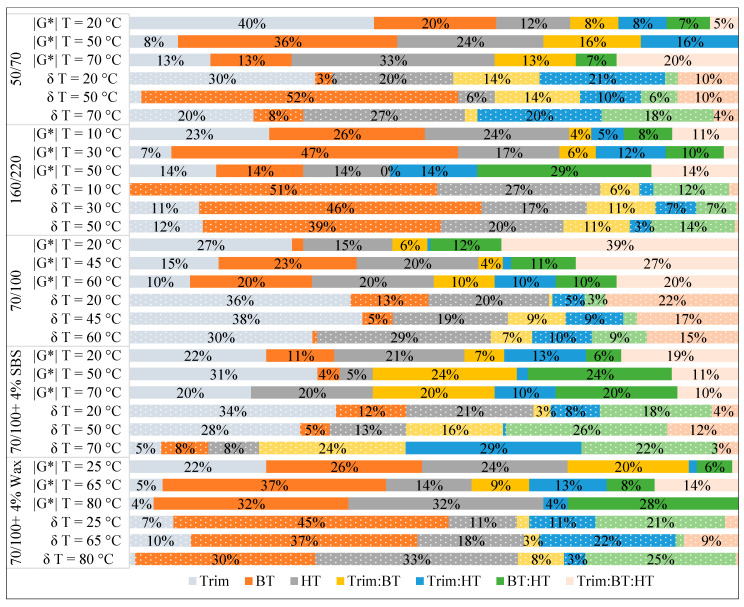
The stacked bar chart shows the effect contributions in % to the variations in |G*| (solid-filled) and *δ* (pattern-filled) at temperatures below and around the SP of tested materials.

In Figure 4 and Figure 5, the stacked bar chart is used to show the contribution of the effects (both main and interaction) to the outcomes (|G*|, *δ*, *TX*, and *δ_TX_*). The percentage contribution of each effect is calculated by dividing each effect by the total of all seven effects in a set of tests, which allows us to compare and estimate the share of the variation that could be attributable to a particular effect across the tested materials. The factors with higher values are the most important, while those with lower values are the least important. The importance of the effects is not independent of the type of bitumen, according to the ranking of their contribution to outcomes. However, the results observed at higher test temperatures tend to be more susceptible to fluctuations in test conditions, particularly for modified bitumen. Even though the three main effects are expected to account for a large portion of the variation, interactions between the components appeared to be statistically significant, particularly in the case of SBS-modified bitumen for *TX* and *δ_TX_* values. However, the significant interaction effect cannot be interpreted without taking into account the main effects involved. The three-way interaction Trim.BT.HT has the most contribution for bitumen type 70/100 (Figure 4 and Figure 5).

## 5. Conclusions

This study used a two-level three-factor factorial design to investigate the effects of the following factors on the measured |G*|, *δ*, *TX*, and *δ_TX_* via DSR: the pre-heating temperature (HT) of bitumen to manufacture the specimens in a mould, the bonding temperature (BT) at which the specimen is bonded onto DSR plates, and the radial trimming operation of the specimen after the DSR plates are loaded with the specimen. Based on the laboratory experiments and statistical analyses, the following conclusions can be drawn:

- The effect of the trimming operation (trimming vs. not trimming) of the specimen is significant when employing PP08, but not PP25. This could be because PP25 is easier to trim than PP08 due to the larger diameter and lower height of the specimen.
- At higher test temperatures when using PP25, the HT and BT contribute more to the variation in the measured outcomes. Since PP25 has a larger surface area, it may be more sensitive to ensuring that the bonding temperature chosen is sufficient for the adhesion of the test specimen to the rheometer plates.
- Aside from the bitumen type 70/100 results, which appeared to be influenced by the individual batch of material used, unmodified binders are less susceptible to fluctuation in the studied parameters than modified ones. The three-way interaction Trim.BT.HT tends to contribute relatively little to the measured parameter variation.
- Interactions between two factors Trim.BT, Trim.HT, and BT.HT contribute more to the fluctuation in *δ* values than *TX* and |G*|.
- The variation in examined factors considerably influenced the repeatability based on data from wax-modified bitumen. However, considering the various testing settings used in this research, the estimated repeatability r precision for outcomes except for wax-modified bitumen is comparable to the values given in EN14770:2023, and it is within repeatability criteria r. When data from wax-modified bitumen are ignored, the ranges for the variables r(*TX*), r(*δ_TX_*), r(|G*|), and r(*δ*) are 0.4–2.1 °C, 0.3–3.1°, 5–31%, and 0.2–3.5°, respectively.
- Overall, it is reasonable to conclude that variations in testing instructions can have a significant impact on DSR-recorded rheological parameters of bitumen. As a result, this study shows that clarifying the testing instructions and emphasising the need for operators to follow them closely can enhance consistency in the test results.

Our findings highlight the importance of clear guidance (instruction) and operators and following them closely to enhance consistency in the test results. The current method EN 14770:2023 can be improved following our findings. The specified bonding temperature (BT) needs to be closely followed or updated to a smaller interval, and a more precise temperature setting (HT) for sample manufacturing can be set. Also, trimming operations could be mandatory to ensure the same amount of substance is tested rather than relying on individual operators’ skills to precisely position the specimen on the centre of the plate to obtain an even bulge around the perimeter. Organising a round-robin (interlaboratory) test on relevant factors to be tested can greatly help in implementing these findings into the next version of the specification (EN).

## Figures and Tables

**Table 1:** Bitumen types and properties.

Material	PEN at 25 °C (0.1 mm) EN 1426	SP (°C) EN 1427	Density at 25 °C (g/cm^3^) ASTM D70
50/70	61	46.8	1.025
160/220	160	41.2	1.000
70/100	85	45.6	1.019
70/100 + 4% SBS	39	67.2	1.015
70/100 + 4% Wax	33	79.0	1.014

**Table 2:** The 8 combinations of the 2^3^ factorial design with two replicates and their testing order.

8 Different Combs.	Randomised Test Order	Contrast Coefficients for Main and Interaction Effects						
		Trim ^1^	BT ^2^	HT ^3^	Trim.BT	Trim.HT	BT.HT	Trim.BT.HT
1	6 and 13	(−1)	(−1)	(−1)	(+1)	(+1)	(+1)	(−1)
2	2 and 4	(+1)	(−1)	(−1)	(−1)	(−1)	(+1)	(+1)
3	1 and 16	(−1)	(+1)	(−1)	(−1)	(+1)	(−1)	(+1)
4	5 and 10	(+1)	(+1)	(−1)	(+1)	(−1)	(−1)	(−1)
5	8 and 12	(−1)	(−1)	(+1)	(+1)	(−1)	(−1)	(+1)
6	9 and 14	(+1)	(−1)	(+1)	(−1)	(+1)	(−1)	(−1)
7	3 and 11	(−1)	(+1)	(+1)	(−1)	(−1)	(+1)	(−1)
8	7 and 15	(+1)	(+1)	(+1)	(+1)	(+1)	(+1)	(+1)

^1^ Trim (Trimming operation) levels: (−1) Yes; (+1) No. ^2^ BT (Bonding Temp.) levels: (−1) SP + 0 °C; (+1) SP + 25 °C. ^3^ HT (Pre-heating Temp.) levels: (−1) SP + 80 °C; (+1) SP + 100 °C.

**Table 3:** Testing plan for capturing the temperature (*TX*) and the related phase angle (*δ_TX_*) at dynamic shear modulus values of 5 MPa, 15 kPa, and 1 kPa.

Material	Specimen 1 (PP08) *TX*, *δ_TX_* (at 5 MPa)	Specimen 2 (PP25) *TX*, *δ_TX_* (at 15 kPa)	Specimen 3 (PP25) *TX*, *δ_TX_* (at 1 kPa)
50/70	20 and 10 °C	50 and 60 °C	70 and 80 °C
160/220	10 and 0 °C	**30** and 40 °C	50 and 60 °C
70/100	**20** and 10 °C	**45** and 55 °C	**60** and 65 °C
70/100 + 4% SBS	**20** and 10 °C	**50** and 60 °C	**70** and 75 °C
70/100 + 4% Wax	**25** and 15 °C	**65** and 75 °C	**80** and 85 °C

Note. Temperatures at which samples were trimmed are shown in bold.

**Table 4:** Analysis of variance for the outcome *T*(|G*| = 5 MPa) for bitumen 70/100.

Factor	Degree Freedom	Sum of Squares	Mean Square	F-Value	*p*-Value (Prob > F)
Trim	1	0.964	0.964	2.491	0.153
BT	1	0.003	0.003	0.009	0.929
HT	1	0.297	0.297	0.768	0.406
Trim.BT	1	0.062	0.062	0.159	0.700
Trim.HT	1	0.013	0.013	0.033	0.861
BT.HT	1	0.188	0.188	0.486	0.505
Trim.BT.HT	1	3.188	3.188	8.243	0.028 *
Residuals	8	3.094	0.387	-	-
Total	15	7.809	-	-	-

* Indicates statistical significance at a 5% level.

**Table 5:** The calculated effects and standard errors from contrast coefficients between combinations for the outcome *T*(|G*| = 5 MPa) for bitumen 70/100.

Factors	Trim	BT	HT	Trim.BT	Trim.HT	BT.HT	Trim.BT.HT
High-level combs. (Y_+1_)	2,4,6,8	3,4,7,8	5,6,7,8	1,4,5,8	1,3,6,8	1,2,7,8	2,3,5,8
Low-level combs. (Y_−1_)	1,3,5,7	1,2,5,6	1,2,3,4	2,3,6,7	2,4,5,7	3,4,5,6	1,4,6,7
$\mathrm{Effects}\;({2.a}_{i}$): Avg.Y_+1_ − Avg.Y_−1_	0.49	−0.03	0.27	−0.12	−0.06	−0.22	−0.89 *
Standard Error (effect) $=\sqrt{\left(\frac{1}{8}+\frac{1}{8}\right)\cdot (\sum_{n=1}^{n=8}\left(\frac{{Diff.\;of\;replicate}^{2}}{2}\;{)}_{n}/8\right)}=\sqrt{\left(\frac{1}{8}+\frac{1}{8}\right)\cdot \left(\frac{2.9}{8}\right)=}$ ± 0.3							

* Indicates statistical significance at a 5% level.

**Table 6:** Estimated means for *TX* and the corresponding *δ_TX_*.

Material	*TX* (°C)			*δ_TX_* (°)		
	*T*_|G*|=5MPa_	*T*_|G*|=15kPa_	*T*_|G*|=1kPa_	*δ*_*T*|G*|=5MPa_	*δ*_*T*|G*|=15kPa_	*δ*_*T*|G*|=1kPa_
50/70	15.5	50.3	70.3	46.2	73.9	83.7
160/220	7.6	39.7	57.3	41.8	71.2	81.8
70/100	13.0	48.2	68.1	45.9	73.8	83.
70/100 + 4% SBS	14.4	55.3	79.3	42.9	65.4	81.1
70/100 + 4% Wax	23.2	70.9	84.2	33.9	42.8	56.8

**Table 7:** Estimated means for |G*| and means for δ.

Material	|G*| (kPa)			*δ* (°)		
50/70	T = 20 °C	T = 50 °C	T = 70 °C	T = 20 °C	T = 50 °C	T = 70 °C
	2434.1	15.8	1.0	50.7	73.7	83.6
160/220	T = 10 °C	T = 30 °C	T = 50 °C	T = 10 °C	T = 30 °C	T = 50 °C
	3544.7	73.7	2.9	43.8	63.4	78.10
70/100	T = 20 °C	T = 45 °C	T = 60 °C	T = 20 °C	T = 45 °C	T = 60 °C
	1638.8	23.5	2.8	52.5	71.9	80.5
70/100 + 4% SBS	T = 20 °C	T = 50 °C	T = 70 °C	T = 20 °C	T = 50 °C	T = 70 °C
	2169.4	27.3	2.7	47.7	61.3	76.3
70/100 + 4% Wax	T = 25 °C	T = 65 °C	T = 80 °C	T = 25 °C	T = 65 °C	T = 80 °C
	4143.2	54.9	2.0	34.8	36.6	52.6

**Table 8:** Estimated effects and standard errors for *TX* and corresponding *δ_TX_* (at ω = 10 rad/s).

Material	Effect	*T*_|G*|=5MPa_	*T*_|G*|=15kPa_	*T*_|G*|=1kPa_	*δ_T_*_|G*|=5MPa_	*δ_T_*_|G*|=15kPa_	*δ_T_*_|G*|=1kPa_
50/70	Trim	0.42 ** ± 0.2	−0.06 ± 0.1	−0.10 ± 0.1	0.21 * ± 0.2	−0.04 ± 0.1	0.02 ± 0.1
	BT	−0.22 * ± 0.2	0.17 * ± 0.1	0.08 ± 0.1	−0.26 * ± 0.2	−0.02 ± 0.1	0.00 ± 0.1
	HT	0.12 ± 0.2	0.14 ± 0.1	0.22 * ± 0.1	0.00 ± 0.2	0.07 * ± 0.1	0.00 ± 0.1
	Trim.BT	−0.08 ± 0.2	0.08 ± 0.1	0.11 ± 0.1	−0.17 ± 0.2	0.01 ± 0.1	0.02 ± 0.1
	Trim.HT	0.07 ± 0.2	0.09 ± 0.1	0.03 ± 0.1	−0.04 ± 0.2	0.08 * ± 0.1	0.06 ± 0.1
	BT.HT	0.08 ± 0.2	−0.01 ± 0.1	−0.05 ± 0.1	0.08 ± 0.2	−0.02 ± 0.1	−0.06 ± 0.1
	Trim.BT.HT	0.06 ± 0.2	−0.01 ± 0.1	−0.12 ± 0.1	0.10 ± 0.2	0.02 ± 0.1	−0.03 ± 0.1
160/220	Trim	0.64 * ± 0.4	−0.09 ± 0.1	0.19 ± 0.3	0.42 ± 0.6	0.14 ± 0.1	0.02 ± 0.1
	BT	0.65 ** ± 0.4	0.59 ** ± 0.1	−0.21 ± 0.3	−1.50 ** ± 0.6	−0.47 ** ± 0.1	−0.16 * ± 0.1
	HT	−0.63 * ± 0.4	−0.17 * ± 0.1	0.35 * ± 0.3	0.60 ± 0.6	0.05 ± 0.1	0.06 ± 0.1
	Trim.BT	−0.17 ± 0.4	−0.09 ± 0.1	−0.11 ± 0.3	0.15 ± 0.6	0.22 * ± 0.1	−0.05 ± 0.1
	Trim.HT	−0.03 ± 0.4	−0.26 ** ± 0.1	0.03 ± 0.3	−0.19 ± 0.6	0.04 ± 0.1	−0.02 ± 0.1
	BT.HT	0.27 ± 0.4	0.31 ** ± 0.1	−0.04 ± 0.3	−0.30 ± 0.6	−0.12 ± 0.1	−0.04 ± 0.1
	Trim.BT.HT	0.17 ± 0.4	−0.044 ± 0.1	−0.16 ± 0.3	0.10 ± 0.6	−0.06 ± 0.1	−0.06 ± 0.1
70/100	Trim	0.25 ± 0.3	0.17 ** ± 0.1	0.18 ** ± 0.1	−0.19 ± 0.3	−0.30 ** ± 0.1	−0.05 ± 0.1
	BT	−0.01 ± 0.3	−0.17 ** ± 0.1	0.00 ± 0.1	0.15 ± 0.3	−0.19 ** ± 0.1	−0.03 ± 0.1
	HT	0.14 ± 0.3	0.22 ** ± 0.1	0.23 ** ± 0.1	−0.18 ± 0.3	−0.07 ± 0.1	−0.06 * ± 0.1
	Trim.BT	−0.06 ± 0.3	−0.02 ± 0.1	−0.12 * ± 0.1	0.05 ± 0.3	−0.11 ** ± 0.1	−0.06 * ± 0.1
	Trim.HT	−0.03 ± 0.3	0.01 ± 0.1	0.10 * ± 0.1	−0.12 ± 0.3	−0.10 * ± 0.1	−0.01 ± 0.1
	BT.HT	−0.11 ± 0.3	−0.11 * ± 0.1	−0.16 ** ± 0.1	−0.06 ± 0.3	−0.05 ± 0.1	−0.03 ± 0.1
	Trim.BT.HT	−0.45 * ± 0.3	−0.30 ** ± 0.1	−0.29 ** ± 0.1	−0.04 ± 0.3	−0.00 ± 0.1	−0.06 ± 0.1
70/100 + 4% SBS	Trim	0.34 * ± 0.2	0.33 ** ± 0.2	0.23 ** ± 0.1	−0.18 ± 0.3	−0.34 ** ± 0.2	0.71 ** ± 0.4
	BT	−0.18 ± 0.2	−0.01 ± 0.2	0.04 ± 0.1	−0.03 ± 0.3	−0.11 ± 0.2	0.59 * ± 0.4
	HT	0.35 * ± 0.2	0.08 ± 0.2	0.17 * ± 0.1	−0.02 ± 0.3	−0.18 ± 0.2	0.31 ± 0.4
	Trim.BT	0.15 ± 0.2	0.24 * ± 0.2	0.35 ** ± 0.1	0.18 ± 0.3	−0.29 * ± 0.2	−0.34 ± 0.4
	Trim.HT	0.22 ± 0.2	0.00 ± 0.2	0.36 ** ± 0.1	0.05 ± 0.3	−0.25 * ± 0.2	−0.90 ** ± 0.4
	BT.HT	−0.12 ± 0.2	0.27 ** ± 0.2	0.42 ** ± 0.1	−0.38 * ± 0.3	−0.53 ** ± 0.2	−0.15 ± 0.4
	Trim.BT.HT	−0.34 * ± 0.2	0.14 ± 0.2	0.16 * ± 0.1	−0.24 ± 0.3	−0.18 ± 0.2	0.40 ± 0.4
70/100 + 4% Wax	Trim	0.653 ± 0.6	0.15 ± 0.4	0.21 ± 0.3	0.11 ± 0.5	0.85 ± 0.9	0.20 ± 0.4
	BT	0.76 * ± 0.6	0.75 ** ± 0.4	−0.66 ** ± 0.3	−1.05 ** ± 0.5	−0.32 ± 0.9	2.09 ** ± 0.4
	HT	0.79 * ± 0.6	0.54 * ± 0.4	1.29 ** ± 0.3	0.04 ± 0.5	−0.75 ± 0.9	−2.43 ** ± 0.4
	Trim.BT	0.65 ± 0.6	0.32 ± 0.4	0.02 ± 0.3	0.38 ± 0.5	0.37 ± 0.9	0.42 ± 0.4
	Trim.HT	0.01 ± 0.6	−0.31 ± 0.4	−0.09 ± 0.3	0.30 ± 0.5	0.81 ± 0.9	−0.08 ± 0.4
	BT.HT	0.11 ± 0.6	−0.11 ± 0.4	−0.09 ** ± 0.3	−0.56 ± 0.5	0.57 ± 0.9	2.10 ** ± 0.4
	Trim.BT.HT	−0.03 ± 0.6	−0.11 ± 0.4	0.09 ± 0.3	0.05 ± 0.5	−0.23 ± 0.9	−0.09 ± 0.4

* and ** indicate statistical significance at 5% and 1% levels, respectively.

**Table 9:** Estimated effects and standard errors on measured |G*| and *δ* at temperatures below and around the SP of tested materials (at ω = 10 rad/s).

Material	Effect	|G*|			δ		
	@Temp.	@ 20 °C	@ 50 °C	@ 70 °C	@ 20 °C	@ 50 °C	@ 70 °C
50/70	Trim	335.6 ** ± 60.3	−0.2 ± 0.4	−0.0 ± 0.0	−0.43 ± 0.2	−0.01 ± 0.1	0.10 * ± 0.0
	BT	−167.5 * ± 60.3	0.9 * ± 0.4	0.0 ± 0.0	−0.04 ± 0.2	−0.26 ** ± 0.1	−0.04 ± 0.0
	HT	101.3 ± 60.3	0.6 ± 0.4	0.1 * ± 0.0	−0.28 ± 0.2	−0.03 ± 0.1	−0.13 * ± 0.0
	Trim.BT	−66.0 ± 60.3	0.4 ± 0.4	0.0 ± 0.0	−0.20 ± 0.2	−0.07 ± 0.1	−0.01 ± 0.0
	Trim.HT	66.1 ± 60.3	0.4 ± 0.4	0.0 ± 0.0	−0.29 ± 0.2	0.05 ± 0.1	0.10 * ± 0.0
	BT.HT	59.5 ± 60.3	0.0 ± 0.4	−0.0 ± 0.0	0.03 ± 0.2	−0.03 ± 0.1	−0.09 * ± 0.0
	Trim.BT.HT	38.8 ± 60.3	−0.0 ± 0.4	−0.0 ± 0.0	0.14 ± 0.2	0.05 ± 0.1	0.02 ± 0.0
160/220	**@Temp.**	**@ 10 °C**	**@ 30 °C**	**@ 50 °C**	**@ 10 °C**	**@ 30 °C**	**@ 50 °C**
	Trim	595.6 ** ± 157.8	−3.5 ± 2.8	−0.1 ± 0.1	0.00 ± 0.6	1.01 ± 0.5	0.21 ± 0.1
	BT	660.8 ** ± 157.8	23. 7 ** ± 2.8	0.1 ± 0.1	−4.26 ** ± 0.6	−4.08 ** ± 0.5	−0.68 ** ± 0.1
	HT	−614.1 ** ± 157.8	−8.4 * ± 2.8	0.1 ± 0.1	2.26 ** ± 0.6	1.52 * ± 0.5	−0.35 * ± 0.1
	Trim.BT	−93.6 ± 157.8	−3.0 ± 2.8	0.0 ± 0.1	0.54 ± 0.6	0.99 ± 0.5	0.19 ± 0.1
	Trim.HT	−138.8 ± 157.8	−5.8 ± 2.8	−0.1 ± 0.1	−0.19 ± 0.6	0.59 ± 0.5	−0.06 ± 0.1
	BT.HT	205.5 ± 157.8	4.8 ± 2.8	0.2 * ± 0.1	−1.05 ± 0.6	−0.58 ± 0.5	−0.24 ± 0.1
	Trim.BT.HT	281.2 ± 157.8	−1.2 ± 2.8	−0.1 ± 0.1	−0.13 ± 0.6	0.03 ± 0.5	0.01 ± 0.1
70/100	**@Temp.**	**@ 20 °C**	**@ 45 °C**	**@ 60 °C**	**@ 20 °C**	**@ 45 °C**	**@ 60 °C**
	Trim	183.0 ± 92.4	1.1 ** ± 0.3	0.1 ** ± 0.0	−1.25 ** ± 0.1	−0.85 ** ± 0.1	−0.40 ** ± 0.0
	BT	−12.8 ± 92.4	−1.7 ** ± 0.3	−0.2 ** ± 0.0	0.44 ** ± 0.1	−0.11 ± 0.1	0.01 ± 0.0
	HT	100.1 ± 92.4	1.5 ** ± 0.3	0.2 ** ± 0.0	−0.68 ** ± 0.1	−0.42 ** ± 0.1	−0.38 ** ± 0.0
	Trim.BT	−39.5 ± 92.4	−0.3 ± 0.3	−0.1 ** ± 0.0	0.02 ± 0.1	−0.21 * ± 0.1	−0.09 ** ± 0.0
	Trim.HT	3.3 ± 92.4	0.1 ± 0.3	0.1 * ± 0.0	−0.18 ± 0.1	−0.21 * ± 0.1	−0.13 ** ± 0.0
	BT.HT	−79.8 ± 92.4	−0.8 ± 0.3	−0.1 ** ± 0.0	0.12 ± 0.1	0.05 ± 0.1	0.12 ** ± 0.0
	Trim.BT.HT	−266.5 * ± 92.4	−2.0 ** ± 0.3	−0.2 ** ± 0.0	0.75 ** ± 0.1	0.37 ± 0.1	0.20 ** ± 0.0
70/100 + 4% SBS	**@Temp.**	**@ 20 °C**	**@ 50 °C**	**@ 70 °C**	**@ 20 °C**	**@ 50 °C**	**@ 70 °C**
	Trim	265.9 ** ± 70.7	1.7 ** ± 0.5	0.2 ** ± 0.0	−1.09 ** ± 0.2	−1.27 ** ± 0.2	−0.34 ± 0.3
	BT	−131.8 ± 70.7	−0.2 ± 0.5	0.0 ± 0.0	0.37 ± 0.2	−0.22 ± 0.2	0.50 ± 0.3
	HT	252.8 ** ± 70.7	0.3 ± 0.5	0.2 ** ± 0.0	−0.67 ** ± 0.2	−0.57 * ± 0.2	−0.54 ± 0.3
	Trim.BT	77.2 ± 70.7	1.3 * ± 0.5	0.2 ** ± 0.0	0.09 ± 0.2	−0.72 ** ± 0.2	−1.56 ** ± 0.3
	Trim.HT	158.8 ± 70.7	−0.1 ± 0.5	0.1 ** ± 0.0	−0.26 ± 0.2	0.02 ± 0.2	−1.87 ** ± 0.3
	BT.HT	−68.2 ± 70.7	1.3 * ± 0.5	0.2 ** ± 0.0	−0.59 * ± 0.2	−1.20 ** ± 0.2	−1.45 ** ± 0.3
	Trim.BT.HT	−227.2 * ± 70.7	0.6 ± 0.5	0.1 ** ± 0.0	0.14 ± 0.2	−0.53 * ± 0.2	−0.22 ± 0.3
70/100 + 4% Wax	**@Temp.**	**@ 25 °C**	**@ 65 °C**	**@ 80 °C**	**@ 25 °C**	**@ 65 °C**	**@ 80 °C**
	Trim	677.1 * ± 255.4	3.7 ± 2.0	0.1 ± 0.1	−0.46 ± 0.5	0.97 ± 0.8	0.16 ± 0.4
	BT	770.3 * ± 255.4	24.8 ** ± 2.0	−0.8 ** ± 0.1	−2.87 ** ± 0.5	−3.57 ** ± 0.8	4.78 ** ± 0.4
	HT	719.0 * ± 255.4	9.5 ** ± 2.0	0.8 ** ± 0.1	−0.71 ± 0.5	−1.68 ± 0.8	−5.38 ** ± 0.4
	Trim.BT	599.6 * ± 255.4	6.4 * ± 2.0	0.0 ± 0.1	0.13 ± 0.5	0.24 ± 0.8	1.23 * ± 0.4
	Trim.HT	39.4 ± 255.4	−8.6 ** ± 2.0	−0.1 ± 0.1	0.69 ± 0.5	2.15 * ± 0.8	0.56 ± 0.4
	BT.HT	174.1 ± 255.4	5.3 * ± 2.0	−0.7 ** ± 0.1	−1.35 * ± 0.5	0.13 ± 0.8	4.00 ** ± 0.4
	Trim.BT.HT	30.8 ± 255.4	−9.3 ** ± 2.0	0.0 ± 0.1	0.14 ± 0.5	0.86 ± 0.8	−0.07 ± 0.4

* and ** indicate statistical significance at 5% and 1% levels, respectively.

## Data Availability

Data available on request from the corresponding author.
